# Identification of selection signatures and genetic diversity in the sheep

**DOI:** 10.1007/s11250-025-04307-9

**Published:** 2025-02-18

**Authors:** Mustafa Karabaş, Onur Yılmaz

**Affiliations:** Faculty of Agriculture Animal Science Department, Aydın Adnan Menderes University, 09020 Aydın, Türkiye

**Keywords:** Integrated haplotype score, Runs of homozygosity, Genetic diversity, Selective sweep

## Abstract

In the study, data obtained from OvineSNP50K SNP chips using the Illumina® iScan platform for Eşme sheep were used. The integrated haplotype score (iHS) and runs of homozygosity (ROH) statistical approaches were used to identify selection signatures. Using the iHS analysis, it was discovered that there are 10 genomic regions and 51 genes on ovine chromosomes 1, 9, 11, and 12 that are under selection. Three genomic regions and 97 genes on ovine chromosomes 6 and 11 were found to be under selection using the ROH analysis. Candidate genes associated with economic and ecological traits were detected using both approaches. Among the genetic diversity parameters considered in this study, the minor allele frequency (MAF), the genetic distance between individuals (D), as well as observed (Ho) and expected heterozygosities (He) values were 0.300, 0.309, 0.388, and 0.390, respectively. The obtained Ho, He and D values indicate a moderate level of genetic diversity. The ratio of polymorphic SNPs (PN) was 0.947, and the average values of F_ROH_ and F_HOM_ were 0.030 and 0.029, respectively. Considering the PN value obtained in the study, it is evident that the SNPs in the population exhibit a high level of polymorphism at 94.7%. While the F_ROH_ value obtained indicates high genetic diversity among the individuals in the present study, the F_HOM_ value suggests that the population is predominantly composed of heterozygous individuals. As a result, evidence indicating genetic advancements have been made for target traits in breeding programs within the population. Additionally, candidate genes suitable for future molecular marker-supported breeding programs have been identified. In addition, a better understanding of the genetic structure and production potential of the population has been achieved. Findings have shown that Eşme sheep are a breed with high meat production potential and strong adaptation abilities.

**Supplementary Information:**

The online version contains supplementary material available at 10.1007/s11250-025-04307-9.

## Introduction

The formation of a shared environment between human and wild animal populations has led to increased interaction between species (Larson and Fuller 2014). These interactions have caused humans to alter the functioning of evolutionary mechanisms in certain wild animal species through the use of knowledge, creativity, and cultural transfer abilities, resulting in the emergence of various domestication processes (Yamamoto et al. 2013). Selection (both artificial and natural), genetic drift, and adaptation are all processes that contribute to changes in genotypic and phenotypic characteristics associated with morphological, behavioral, and production traits (Diamond 2002; Mignon-Grasteau et al. 2005; Zeder 2012; Lye and Purugganan 2019; Frantz et al. 2020; Saravanan et al. 2020).

Evolutionary processes can only be effective in the presence of hereditary genetic differences created by chance mutations in living organisms (Whitehead and Crawford 2006). Beneficial mutations occurring in wild and domestic animals tend to rapidly fixate in the population because they have the chance to be transferred to the next generation (Cui and Yuan 2018). On the contrary, harmful mutations tend to be eliminated from the population because they cause negative effects (Marsden et al. 2016). Beneficial mutations spread along with corresponding neutral mutations on the genome during the fixation process in the population, due to crossing-over, natural selection, genetic drift, or recombination. In this case, known as the hitchhiking effect, the beneficial mutation becomes fixed in the population through selective sweep, resulting in a decrease in genetic variation around it. As a result of decreasing genetic variation caused by selective sweeps, genomic footprints, known as selection signatures, appear on the genome (Kaplan et al. 1989; Stephan 2019). This causes the formation of genomic footprints during both the fixation and sorting processes, making it easier to detect these signatures (Charlesworth and Charlesworth 2018). Selection signatures may provide important information about the direction and functioning of natural and artificial selection processes that have occured during the development of farm animal breeds. This information has made it possible to identify genomic footprints of both human-mediated artificial (e.g. production traits) and natural (e.g. adaptive traits) selection (Kim et al. 2013; Cheruiyot et al. 2018).

In addition, demographic processes can also lead to misleading selection signatures. For example, the incorporation of new genomic regions into populations that have recently experienced significant hybridization or migration can be regarded as a selective sweep and defined as a selection signature (Oleksyk et al. 2010). For this reason, it is crucial to have a thorough understanding of the evolutionary history of the population in which selection signatures are identified. Additionally, adjustments must be made to minimize false signal noise in the selection signature identification methods employed. In recent years, advancements in molecular genetic techniques and bioinformatics have enabled the detection of selection signals within and between populations using various genotyping and sequencing technologies combined with statistical methododologies (Gurgul et al. 2018; Almeida et al. 2019; Moradian et al. 2020). Among the within-population methods, the Integrated Haplotype Score (iHS) and Runs of Homozygosity (ROH) approaches are widely used to provide information about long homozygous haplotype regions formed around mutations under selection and, therefore, to detect selection signatures (Liu et al. 2021).

The iHS method is used to identify recent selective sweeps by comparing the degradation rates of haplotype homozygosity between selected and alternative alleles within a locus. By incorporating alternate allele data into the computations, this approach provides insights into regional recombination and reduces the likelihood of detecting false selection signals (Voight et al. 2006; Sabeti et al. 2007). The utilization of genomic data through the ROH approach yields significant insights into the levels of kinship present within the population (McQuillan et al. 2008). Nonetheless, it is possible to define homozygous haplotype models with ROH islands formed as a result of selective drift (Purfield et al. 2017).

As a result, selection signature approaches have become extensively employed in the livestock genetics research to identify genomic regions and genes that are experiencing positive selection (Saravanan et al. 2020). In populations subject to no (on less intensive) selection, mutations that confer survival advantages are anticipated to be subject to natural selective pressures, whereas mutations that pertain to economically important production traits are expected to be subject to artificial selection (Onzima et al. 2018). Understanding population structures through selection signature approaches makes a significant contribution to achieving goals such as preserving genetic diversity, increasing adaptation, and ensuring the long-term robustness of species or populations. This approach allows for the prediction of adaptation potential and evolutionary responses (Abied et al. 2020).

Türkiye, one of the most significant transit points between Asia and Europe, is also a crucial center for domestication (Stiner et al. 2022; Özmen and Karaman 2023). Most of the local sheep breeds raised in Türkiye have undergone unsystematic crossbreeding by breeders for centuries, leading to their natural adaptation to various environmental conditions. Türkiye possesses a significant range of genetic variation, which is evident in the various breeds of animals such as cattle, sheep, and buffalo. This is due to its location at a multicultural and intercontinental intersection. As a result, 45 breeds of sheep have been recognized as national breeds in Türkiye (GDAR 2011). The sheep population in Türkiye is diverse and includes many local ecotypes that differ from the 45 known national breeds in certain characteristics. These genotypes are widespread, particularly in the Eastern and Southeastern Anatolia regions. Sheep make up the majority of the approximately 57 million ovines in Türkiye, with 45 million heads (FAOSTAT 2022). The Eşme sheep breed, one of the local sheep breeds of Western Anatolia, originated from a combination of different local sheep breeds. Within the framework of breeder demands and expectations, as well as field observations and phenotypic data collected, a modern breeding organization was established in 2007 with the goal of enhancing the fertility and lamb development characteristics of the Eşme sheep breed population. Scientific findings derived from production characteristics and their genetic parameters, identification of the population structure using molecular genetic markers, screenings for specific gene regions, and genome-wide relationship analyses have led to the official registration of the Eşme breed as a national breed in 2020. The Eşme sheep has become an important breed in Western Anatolia and its surrounding areas due to its significant potential in terms of fertility and lamb growth characteristics (Cemal et al. 2019; Karaca et al. 2019; Yılmaz et al. 2019; Yilmaz et al. 2022).

The aim of this study was to understand the genetic structure and evolutionary history of the Eşme sheep breed, as well as to examine the genetic diversity, adaptive potential, and production characteristics of the population by identifying selection signaturesresulting from both natural and artificial selection processes. Within the scope of the study, genotypic data were analyzed using the integrated haplotype score (iHS) and runs of homozygosity (ROH) statistical approaches, and gene regions under selection within the population were identified. These analyses aim to provide valuable information for conserving the genetic diversity of Esme sheep, enhancing their adaptability, and optimizing their production potential. Additionally, the data obtained on the evolutionary processes and genetic parameters of Esme sheep are intended to serve as an important reference for the long-term sustainability of the breed and to contribute to modern breeding programs.

## Material and method

### Animal material, DNA isolation and genotyping of DNA samples with 50 K SNP chip

The animal material used in this study consisted of 527 head of Eşme sheep bred in 11 elite flocks within the Eşme district of Usak province (performed under project number: TUBITAK-KAMAG 109G014). Blood samples were collected from the jugular vein into tubes containing K3EDTA. The salt precipitation method was utilized to isolate DNA from the collected blood samples (Miller et al. 1988; Montgomery and Sise 1990). The assessment of both the quantity and quality of DNA after DNA isolation was conducted using a NanoDrop 2000 (Thermo Scientific, USA). Based on the assessed quality and quantity of DNA, high concentrations were diluted with TE buffer to achieve a desired concentration of 50 ng/μl. DNA samples with low concentrations were precipitated using ethanol, and the resulting precipitate was resuspended to a concentration of 50 ng/μl. Single nucleotide polymorphism (SNP) genotyping was performed using the Illumina Ovine50K SNP chip, which consists of 54,241 SNPs according to standard protocols of the Agricultural Biotechnology and Food Safety Application and Research Center at the Aydin Adnan Menderes University.

### Quality control of genotype data

The PLINK v1.9 software was utilized to conduct quality control and processing of SNP genotype data (Purcell et al. 2007). Non-autosomal loci, SNPs with a minor allele frequency of less than 0.05, SNPs with a call rate of less than 90%, and samples with an SNP genotyping success rate of less than 90% were excluded from the analysis. After the filtering process, 45,943 SNPs defined in 514 sheep were used in the study.

### Genetic diversity statistics

Minor allele frequency (MAF), proportion of polymorphic SNPs (PN), average proportion of alleles shared between two individuals (D_ST_), observed heterozygosity (Ho) and expected heterozygosity (He) values were calculated using PLINK v1.9 software (Purcell et al. 2007). MAF values were divided into 7 different classes as 0–0.01, 0.01–0.05, 0.05–0.1, 0.1–0.2, 0.2–0.3, 0.3–0.4 and 0.4–0.5. The proportion of polymorphic SNPs (PN) value was calculated as the ratio of SNPs with MAF value greater than 5%. The D_ST_ value was obtained with the following formula:

$${D}_{ST}=\frac{{IBS}_{2}+\left(\text{0,5}*{IBS}_{1}\right)}{N}$$

where IBS_1_ and IBS_2_ represent the number of loci that share either 1 or 2 alleles identical by state (IBS), respectively, and N indicates the total number of loci. The average pairwise genetic distance (D) value was calculated using the following formula:

$$D=1-{D}_{ST}$$

### Integrated Haplotype Score (iHS) analysis

The iHS test was performed to detect haplotype patterns created on the genome by recent selective sweeps (Voight et al. 2006). The ancestral and derived allele information required for this analysis is not yet known in sheep. For this reason, two different approaches were used to assign allele states. In the first approach, the alleles with the highest frequency were defined as ancestral alleles. In the second approach, the status of alleles was assigned 100 times depending on chance (Kim et al. 2016). Haplotype phasing and imputation were performed using default settings in Beagle 5.1 software (Browning et al. 2018). The iHS values for each SNP were calculated using the “rehh” package program in R (Gautier and Vitalis 2012). Standardized iHS values were calculated using the following formula:

$$iHS=\frac{\text{ln}\left(\frac{{iHH}_{A}}{{iHH}_{D}}\right)-E\left[\text{ln}\left(\frac{{iHH}_{A}}{{iHH}_{D}}\right)\right]}{SD\left[\text{ln}\left(\frac{{iHH}_{A}}{{iHH}_{D}}\right)\right]}$$

iHH_A_ and iHH_D_ values represent the integrated EHH scores calculated for the ancestral and derived alleles, respectively. SNPs with a |iHS| value > 3.2 (top 1%) were accepted as extreme (Voight et al. 2006). Non-overlapping 500-kb genomic regions with 10 SNPs, of which at least five are extreme SNPs, have been defined under selection for selection as a single SNP in the genome (Qanbari et al. 2011; Saravanan et al. 2021a, b).

### Runs of Homozygosity (ROH) analysis

ROH analysis was performed in the "detectRUNS" R-package using the sliding-window based method (Biscarini et al. 2018). A minimum threshold of 40 to 50 SNPs in selection signature studies is a common standard in runs of homozygosity (ROH) analyses. To ensure consistency with previous studies and to minimize technical and genotyping errors, the minimum number of SNPs in the sliding window and ROH segment was established at 41 in the present study, and was calculated as suggested by Purfield et al. (2012);

$$L=\frac{{\text{log}}_{e}\frac{a}{{n}_{s}{n}_{i}}}{{\text{log}}_{e}\left(1-het\right)}$$

where, "*a*" is the percentage of false positive ROHs, set at 0.05 in the study. “*n*_*s*_” represents the number of genotyped SNPs per individual, “*ni*” the number of genotyped individuals, and “*het*” the average heterozygosity. A maximum of 1 heterozygous and 2 missing genotypes are allowed in each window. The gap between consecutive SNPs and the minimum ROH length is defined as 1 Mb. The minimum SNP density was determined as at least 1 SNP per 100 kb. ROHs were divided into 5 different groups according to their length: 1–5 Mb, 5–10 Mb, 10–20 Mb, 20–40 Mb and > 40 Mb, respectively (Saravanan et al. 2021a, b). The number of ROHs and the average length of ROHs were calculated for each class. The ratio of each SNP to one ROH was calculated. SNPs in a run of homozygosity (ROH) that is present in at least 10% of the animals in the population were considered indicative of a selective sweep (Cesarani et al. 2019).

### Genomic related inbreeding coefficients

The ROH-based inbreeding coefficient (F_ROH_) was calculated using the formula provided by McQuillan et al. (2008).

$${F}_{ROH}=\frac{\sum {L}_{ROH}}{{L}_{genome}}$$

where, *∑L*_*ROH*_ represents the sum of the lengths of all ROHs detected in an individual, while *L*_*genome*_ refers to the total length of the genome covered by SNPs.

The inbreeding coefficient (F_HOM_) based on the observed and expected number of homozygotes was calculated using the following formula in the PLINK v1.9 software (Purcell et al. 2007).

$${F}_{HOM}=\frac{O-E}{L-E}$$

*O* represents the number of observed homozygosity, *E* represents the expected number of homozygosity, and *L* represents the number of genotyped autosomal SNPs. The Pearson correlation coefficient was calculated between F_ROH_ and F_HOM_.

### Explanation and functional analysis of important genomic regions

When adapting the Oar v4.0 genome assembly to the ARS-UL_Ramb_V2.0 genome assembly, a 2 Mb shift is observed between these two maps. Although this situation does not cause problems in the identification of SNPs in gene regions, it makes it difficult to determine the current gene information within gene regions. In addition, some regions were frozen in the ARS-UL_Ramb_V2.0 genome assembly, and these regions were incorporated into another gene family. This caused some difficulties in identification. Considering this situation, both genome assemblies were taken into account to ensure accurate evaluation in the study. Enrichment analysis of genes was performed using Enrichr software (Chen et al. 2013). QTLs overlapping with genomic regions were detected using the sheepQTL database (https://www.animalgenome.org/cgi-bin/QTLdb/OA/index).

## Results

### Genetic diversity, Runs of Homozygosty (ROH) distribution and genomic inbreeding

**Fig. 1:**
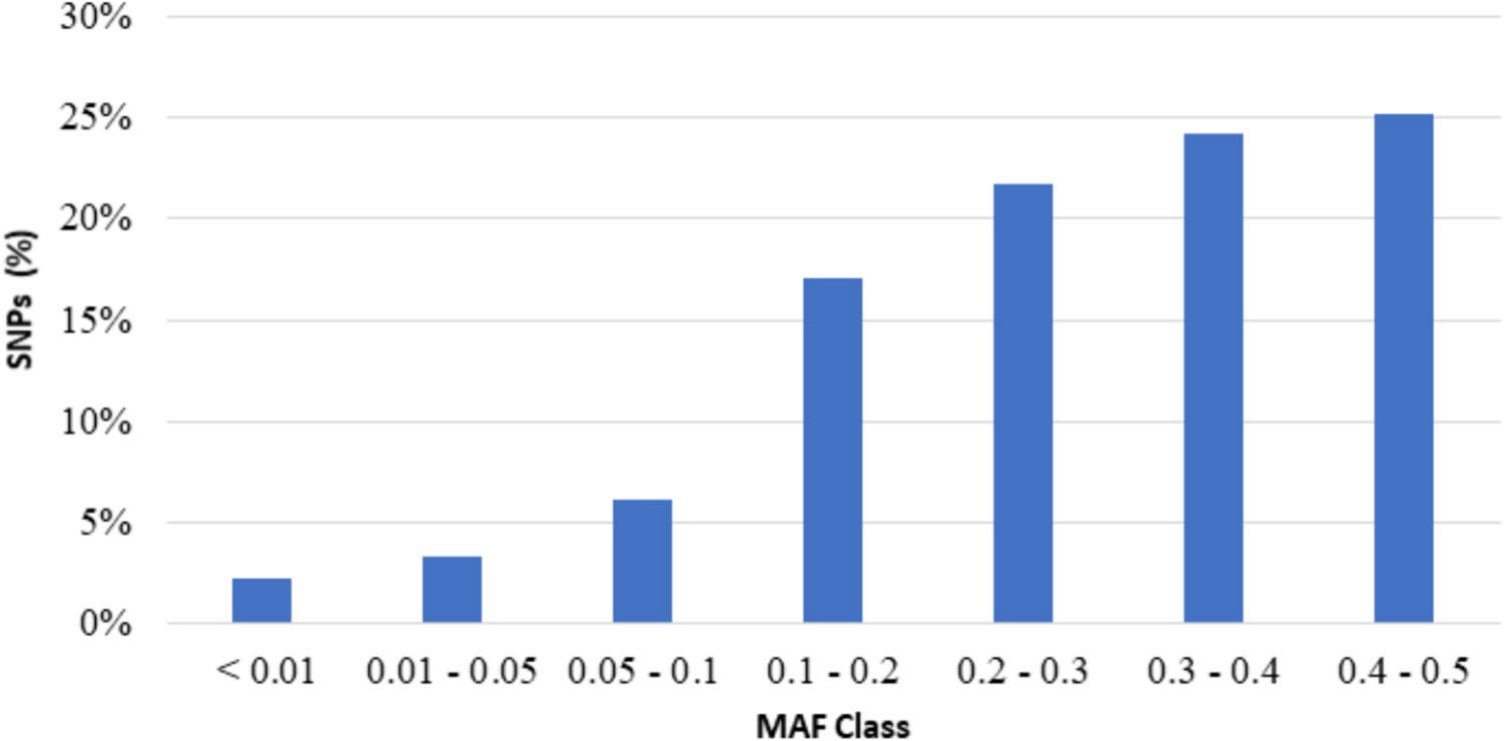
SNPs included in the MAF classes

SNPs included in the MAF classes are shown in Fig. 1.

When examining Table 1, it is notable that the observed heterozygosity value (Ho = 0.388 ± 0.111) was lower than the expected heterozygosity (H_E_ = 0.390 ± 0.109). The mean pairwise genetic distance (D) value, which was used as an indicator of the homogenity among individuals in the population, was calculated to be 0.309 ± 0.016. In terms of *F*_HOM_ value, it was observed that 340 individuals (66.15%) had negative values, while 174 individuals (33.85%) had positive values. A strong and positive correlation coefficient (r = 0.901, p < 0.01) was revealed between F_ROH_ and F_HOM_.

**Table 1:** Parameters of genetic diversity and inbreeding coefficients in the Eşme sheep population (mean ± standard deviation)

MAF	${\text{P}}_{\text{N}}$	${\text{H}}_{\text{O}}$	${\text{H}}_{\text{E}}$	D	F_ROH_	F_HOM_
0.300 ± 0.123	0.947	0.388 ± 0.111	0.390 ± 0.109	0.309 ± 0.016	0.030 ± 0.043	0.029 ± 0.034

MAF: minor allele frequency, PN: the proportion of polymorphic SNPs, Ho: observed heterozygosity, He: expected heterozygosity, D: average pairwise genetic distance, F_ROH_: ROH based inbreeding coefficient, F_HOM_: Inbreeding coefficient based on observed and expected number of homozygosity

Information on ROH based on classes is shown in Table 2. In terms of chromosomes, the highest and lowest number of ROH was observed on chromosome 1 (n = 380) and chromosome 24 (n = 70), respectively (Table S1).

**Table 2:** Summary of the characteristics of runs of homozygosity (ROH) and genomic inbreeding coefficients based on runs of homozygosity (F_ROH_) under different ROH classes

ROH Class (Mb)	ROH		Mean lenght (Mb)	F_ROH_	
	Number	%		Mean	SD
1–5	1621	40.97	3.41	0.0043	0.0037
5–10	1144	28.91	7.05	0.0063	0.0072
10–20	717	18.12	14.03	0.0078	0.0117
20–40	385	9.73	26.62	0.0080	0.0166
> 40	90	2.27	54.01	0.0038	0.0134

F_ROH_: ROH based inbreeding coefficient

The highest average F_ROH_ coefficient was determined in the 20–40 Mb class. It is noteworthy that most of the ROHs are in the 1–10 Mb class, while the highest number of ROHs is observed in the 1–5 Mb class.

### Selection signatures detected by the iHS approach

**Fig. 2:**
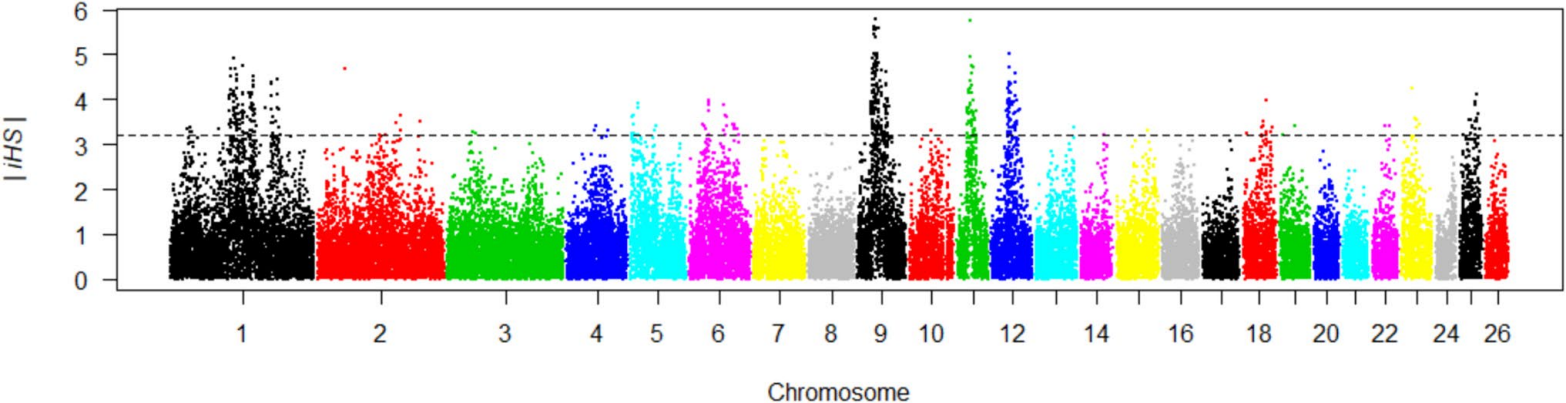
The distribution of |iHS| values across the autosomal genome of the Esme sheep breed

The same results were obtained for the two approaches used to assign ancestral alleles in the iHS analysis. The distribution of |iHS| values across the autosomal genome is shown in Fig. 2.

Ten genomic regions clustered with 65 extreme SNPs (|iHS|> 3.2) on chromosomes 1, 9, 11, and 12 were found to be under selection (Table 3).

**Table 3:** Selection signature detected by the iHS approach

Chr	Region Size (bp)	nSNP	Peak SNP	SNP Position (bp)	|iHS| Value	Genes	No. of Genes
1	150,000,000‐150,500,000	14	OAR1_150005378.1	150,005,378	4.76	-	0
9	31,500,000‐32,000,000	10	OAR9_31965185.1	31,965,185	4.59	LOC105609190, LOC121820332, LOC105609191, CEBPD, SPIDR, LOC121820334, LOC105609193	7
	33,000,000‐33,500,000	10	OAR9_33350205.1	33,350,205	4.19	SNTG1, LOC114116415	2
	34,500,000‐35,000,000	10	OAR9_34630051.1	34,630,051	5.61	ATP6V1H, RGS20, TCEA1, LYPLA1, MRPL15, LOC121820308, SOX17, LOC101114620	8
	37,000,000‐37,500,000	13	s08861.1	37,181,376	5.78	FAM110B, UBXN2B, LOC101116841, SDCBP, NSMAF	5
	39,000,000‐39,500,000	11	OAR9_39334819.1	39,334,819	4.65	CHD7, LOC121820337, LOC114116364, LOC114116365, LOC101111378	5
	43,000,000‐43,500,000	10	OAR9_43354268.1	43,354,268	3.97	LOC101113419, TRNAS-GGA, DNAJC5B, LOC114116545, LOC114116546, TRNAA-AGC, TRIM55, CRH, RRS1, ADHFE1, VXN	11
	48,500,000‐49,000,000	11	OAR9_48558145.1	48,558,145	4.66	KCNB2	1
11	29,500,000‐30,500,000	20	s42356.1	29,798,175	5.76	SHISA6, LOC114116871, DNAH9, ZNF18, MAP2K4, LOC101114791	6
12	39,000,000‐39,500,000	13	OAR12_39089697.1	39,089,697	5.03	AADACL4, LOC114117400, LOC114117294, LOC106991466, DHRS3, VPS13D	6

Chr = Chromosome Number OAR = *Ovis aries*, nSNP = number of SNPs in each iHS signature region

The SNP named s08861.1, which had the highest |iHS| value (5.78), was observed in the genomic region of 37.0–37.5 Mb on chromosome 9. A total of 51 genes were identified in the genomic regions defined as being under selection.

### Selection signatures detected by the ROH approach

**Fig. 3:**
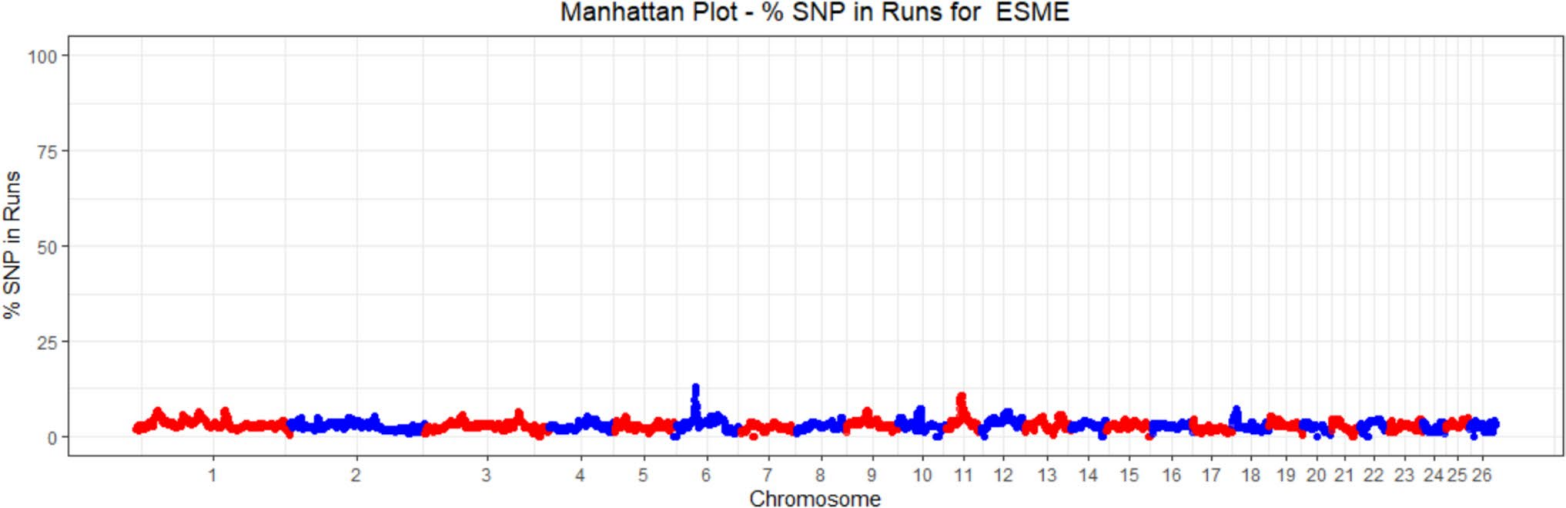
Manhattan graph of the ROH distribution by chromosomes

The distribution of the proportion of each SNP in an ROH across the autosomal genome is shown in Fig. 3. In the ROH analysis, three genomic regions, including 75 SNPs on chromosomes 6 and 11, were identified as selection signatures (Table 4).

**Table 4:** Selection signature detected by the ROH approach

Chr	Region Size (bp)	ROH Length (bp)	Start_SNP	End_SNP	nSNP	Genes	No. of Gene
6	40,311,379–42930840	2,619,461	OAR6_40311379.1	OAR6_42930840.1	41	KCNIP4, LOC101104829, LOC114115434, TRNAW-CCA, ADGRA3, LOC121819870, LOC101105132, GBA3	8
11	26,956,590–27412690	456,100	OAR11_26956590.1	OAR11_27412690.1	4	DNAH2, KDM6B, TMEM88, NAA38, LOC101105010, CHD3, LOC114117094, RNF227, KCNAB3, TRAPPC1, CNTROB, GUCY2D, ALOX15B, LOC101105671, LOC101121438, ALOX12B, ALOXE3, TRNAK-UUU, TRNAQ-CUG, TRNAL-UAG, TRNAR-UCU, HES7, LOC105607806, TRNAG-GCC, LOC114117043, TRNAS-CGA, TRNAT-AGU, PER1, VAMP2, TMEM107, LOC114117047, LOC121820691, TRNAW-CCA, TRNAS-GCU, TRNAT-AGU, TRNAI-AAU, BORCS6, AURKB, TRNAW-CCA, TRNAG-UCC, TRNAD-GUC, TRNAP-CGG, TRNAT-AGU, TRNAS-AGA, TRNAI-AAU, CTC1, PFAS	47
	27,654,920–30218516	2,563,596	OAR11_27654920.1	s49530.1	30	MYH10, LOC114117092, LOC121820692, CCDC42, MFSD6L, PIK3R6, PIK3R5, NTN1, LOC121820582, STX8, TRNAE-UUC, LOC114116874, CFAP52, LOC121820583, USP43, TRNAW-CCA, DHRS7C, GSG1L2, GLP2R, RCVRN, GAS7, TRNAY-GUA, MYH13, LOC121820584, LOC121820585, MYH8, MYH4, MYH1, MYH2, MYH3, LOC121820586, LOC101113508, ADPRM, TMEM220, LOC101114033, TMEM238L, PIRT, SHISA6, LOC114116871, DNAH9, ZNF18, MAP2K4	42

A total of 97 genes were identified in three genomic regions, defined as selective sweep.

### Common selection signatures between IHS and ROH approaches and overlapping QTLs with these methods

10 genomic regions were identified in the IHS analysis, and 3 genomic regions were identified in the ROH analysis. The iHS approach detected an overlap between the 29.5–30.5 Mb region on chromosome 11 and the ROH approach's 27,654,920–30,218,516 bp region on the same chromosome. It has been determined that there are five genes (*SHISA6, LOC114116871, DNAH9, ZNF18, MAP2K4*) in this shared genomic region.

Because of the iHS and ROH analysis, it was determined that the genomic regions defined overlap with a total of 111 QTL, 66 and 45, respectively (Table S2). It was revealed that the identified QTLs were associated with 53.15% meat and carcass, 22.52% milk, 9.91% health, 9.01% production, 1.80% reproduction, and fleece and external structure characteristics (Table S3). The discovery of the highest overlap in QTLs associated with meat and carcass characteristics was considered a significant finding.

### Enrichment analysis of genes under selection

Gene ontology (GO) biological process, GO molecular function, GO cellular component, and Kyoto encyclopedia of genes and genomes (KEGG) gene pathways were determined. The genes obtained for iHS and ROH analysis were statistically significantly enriched (p < 0.05). It was determined that 51 genes identified in the iHS approach were found to be enriched in a total of 26 GO terms and 1 KEGG pathway (Table S4). The identified genes were mainly enriched in terms of *heart morphogenesis (GO: 0003007), regulation of neurotransmitter receptor activity (GO: 0099601), ionotropic glutamate receptor binding (GO: 0035255), MAP kinase kinase activity (GO: 0004708), glutamate receptor binding (GO: 0035254), and nuclear chromosome (GO: 0000228)*. However, the signaling of epithelial cells in *Helicobacter pylori* infection was included in the KEGG pathway. A total of 30 GO terms and 4 KEGG pathways were found to enrich 97 genes identified using the ROH approach (Table S5). The identified genes were mainly enriched in the following terms in the GO database: *actin-myosin filament sliding (GO: 0033275), muscle filament sliding (GO: 0030049), lipoxygenase pathway (GO: 0019372), linoleic acid metabolic process (GO: 0043651), muscle contraction (GO: 0006936), protein serine/threonine/tyrosine kinase activity (GO: 0004712), potassium channel regulator activity (GO: 0015459), muscle myosin complex (GO: 0005859), myosin filament (GO: 0032982), and myofibril (GO: 0030016).* Simultaneously, it has been found that genes are involved in *SNARE* (*phototransduction, soluble N-ethylmaleimide-sensitive factor attachment protein receptors*) interactions in vesicular transport, purine metabolism, and arachidonic acid metabolism KEGG pathways.

## Discussion

In this study, the aim was to determine the genomic regions and genes under selection in Eşme sheep breed by using an integrated haplotype score (iHS), and Runs of Homozygosity (ROH). The rapid increase in the frequency of mutations useful in natural and artificial selection processes in the population decreases genetic variation in the connected regions around the mutation and formation of long homozygous haplotype patterns (Sabeti et al. 2002; Pritchard et al. 2010). However, natural or human-induced demographic events can create genomic footprints that can mimic selection signatures (Oleksyk et al. 2010). For this reason, information about the history and structure of the population being studied is critical in the evaluation of the signals obtained with selection signature approaches. Accordingly, to better evaluate the selection patterns defined in our study, parameters used to estimate the level of genetic diversity in the population were used.

Minor allele frequency (MAF) refers to the frequency of the less common variant of a specific single-nucleotide polymorphism (SNP) in a population. It is expressed as a percentage of the total number of alleles for a SNP and can range from 0 to 100. MAF is an essential measure in genetic association studies. It plays a crucial role in determining the study's power and the probability of detecting an association between a SNP and a phenotype. Additionally, MAF is also used in selection signature analysis to identify genomic regions that have undergone recent positive selection in a population. The distribution of minor allele frequencies obtained from this study was similar to values that were reported for the Sarda sheep breed (Cesarani et al. 2019). The obtained Ho and He values indicate a moderate level of genetic diversity in the studied population. This reveals that the genetic structure of the population varies at a certain level, and the rate of homozygosity is lower than heterozygosity. The mean Ho, and He values obtained in the study were found to be very close to the values reported for Pinzirita sheep (Mastrangelo et al. 2014).

Considering the value obtained in the study, it is evident that the SNPs in the population exhibit a high level of polymorphism at 94.7%. High average pairwise genetic distance (D) values may indicate the existence of genetically distinct subgroups or long-separated populations. The D value obtained from the study indicates a moderate genetic difference, as do the Ho and He values observed in the population under examination. The proportion of polymorphic SNPs (PN) and average pairwise genetic distance (D) values obtained from this study were similar to values reported for Changthangi sheep raised in India (Saravanan et al. 2021a, b). While the F_ROH_ value obtained indicates high genetic diversity among the individuals in the present study, the F_HOM_ value suggests that the population is predominantly composed of heterozygous individuals. The average F_ROH_ value was found to be similar to values reported for Bergamasca (0.026), Istrian Pramenka (0.031), and Biellese (0.034) sheep in their study on Italian sheep breeds (Mastrangelo et al. 2018). This may be attributed to the use of similar chip or analytical methods in previous studies, as well as the fact that the animals used as subjects were raised under comparable breeding conditions to those of the Esme sheep breed. The strong correlation between F_ROH_ and F_HOM_ obtained in the study is consistent with the study conducted in meat sheep (Purfield et al. 2017). The size and distribution of ROHs provide important information about population structure and selection processes (Peripolli et al. 2017). The high number of ROHs obtained between 1 and 5 Mb in the study is consistent with previous studies in sheep (He et al. 2020), pigs (Xie et al. 2019), and cattle (Saravanan et al. 2021a, b). However, the number of ROH > 10 Mb was found to be higher than some studies in sheep (Saravanan et al. 2021a, b; Li et al. 2022). When evaluated together with the F_ROH_ value, these findings generally indicate that the inbreeding between individuals in the population is low, but the effects of inbreeding have also been seen recently.

However, ROHs are not only formed as a result of inbreeding, but also provide information about demographic events and selective sweep. The incidence of ROHs may indicate selective sweeps in the population (Gorssen et al. 2021). Accordingly, in this study, three genomic regions with shared ROHs were defined as selection signatures in at least 10% of individuals. The iHS approach compares the rates of degradation in haplotype homozygosity around ancestral and derived alleles and aims to detect recent selective sweeps (Voight et al. 2006). The findings obtained from the two approaches used to assign ancestral alleles in the iHS analysis in the study were consistent with the literature (Kim et al. 2016). The study revealed that 10 genomic regions, characterized by a certain SNP density and clustering of extreme |iHS|> 3.2 values, are under selection. In the present study, genes under positive selection (rs s08861.1) in Eşme sheep were identified on Chr 9 in the 37–37.5 Mb region (|iHS| value = 5.78) of the autosome. SNPs identified in this region include *FAM110B, UBXN2B, LOC101116841, SDCBP,* and *NSMAF*. It has been reported that these SNPs are associated with meat and carcass traits such as adiposity, sensory and anatomical characteristics (Lee et al. 2013; Li and Kim 2015; Li et al. 2017; Hay and Roberts 2018; de las Heras-Saldana et al. 2020; Naserkheil et al. 2020; Srikanth et al. 2020; Srivastava et al. 2020; Takeda et al. 2020; Naserkheil et al. 2021; Xu et al. 2021; Fonseca et al. 2022); health traits such as resistance to temperature stress, mastitis, and diseases (Kommadath et al. 2014; Chen et al. 2015; Minuti et al. 2015; Smith et al. 2019; Deng et al. 2020; Li et al. 2020; Naserkheil et al. 2020; Brunes et al. 2021; Londoño-Gil et al. 2021; Ruan et al. 2021; Haire et al. 2022); production traits such as growth, feed intake, and feed conversion (Weng et al. 2016; Seabury et al. 2017); reproductive traits such as female fertility and age at calving (Fortes et al. 2012; Gaddis et al. 2017; Mota et al. 2017; Silva et al. 2020; Oliveira et al. 2021; Fonseca et al. 2022) and appearance traits such as coat color (Senczuk et al. 2020; Chhotaray et al. 2021). As the most important selection criteria in the Eşme population, a breeding program is being conducted to target production, reproduction, and meat and carcass characteristics. This program focuses on traits such as fertility, birth weight, weaning weight, and meat quality. The phenotypic data obtained from these breeding programs reveal that significant genetic progress has been made regarding the targeted economic characteristics. The selection signature analysis conducted in the presented study provides genomic evidence that mutations related to production, reproduction, meat, and carcass characteristics are under positive selection. This supports the genetic progress achieved in the breeding program of the Eşme breed sheep population. On the other hand, in the study, it was also defined that mutations associated with health characteristics such as disease susceptibility, parasite resistance, immune capacity, heat adaptation were under positive selection. This finding indicates that the breeding program for the Eşme sheep breed takes into account both economic characteristics and the ability to adapt to ecological conditions in their selection practices. This demonstrates that by refraining from intervening in the extensive breeding practices of the Eşme breed, mutations associated with ecological traits continue to hold significance.

Genomic regions and genes identified as under selection using iHS and ROH approaches have been associated with various ecological and economic traits in studies conducted on livestock. As a result of the obtained associations, an important finding is that the *CEBPD* gene region located in the 31.5–32.5 Mb region on chromosome 9, which is known to be under selection, can serve as a significant genetic marker in selection applications conducted in breeding studies focusing on fertility and lamb development characteristics in the Eşme breed over the course of several years. As a matter of fact, Trukhachev et al. (2016) found that polymorphisms in the *CEBPD* gene region were associated with body parameters in sheep. These polymorphisms were also suggested as markers for breeding programs aimed at improving meat production. There are also reports that the same gene region is effective in temperature stress response in chickens (Park et al. 2019) and fish (Kassahn et al. 2007).

## Conclusion

With iHS approach, it has been determined that there are many candidate genes associated with production, reproduction, meat and carcass characteristics in genomic regions that have recently been identified as under selection in this study. When the literature is examined, polymorphisms identified in these candidate genes have been proposed as molecular markers for important economic characteristics such as body size, body parameters, and meat quality in many studies. Therefore, the findings obtained from the study provide important predictions for molecular marker-asissted breeding programs that may be created in the Eşme population in the future.

Considering the distribution of economic and ecological characteristics associated with the mutations in the genomic regions and genes found to be under selection in the studied population, as well as the overlap of QTLs in the genomic regions and gene pathways, it can be concluded that the Eşme sheep breed has a high potential for meat production and adaptation ability.

This study has made significant contributions to a better understanding of the genetic structure and production potential of Eşme sheep. The results obtained from the study show that breeding programs carried out in the population are planned in accordance with the socio-economic structure of the region and animals with high lamb production potential are selected as breeders under extensive conditions. On the other hand, this accurate breeding selection indicates that genetic progress has been achieved regarding the target economic characteristics. In addition, the identification of genes with mutations that may be beneficial or harmful related to economic and ecological characteristics in the study prepared the basis for molecular marker-assisted breeding plans that can be carried out in the future.

## Supplementary Information

Below is the link to the electronic supplementary material.

Supplementary file1 (DOCX 14 KB)

Supplementary file2 (DOCX 35 KB)

Supplementary file3 (DOCX 14 KB)

Supplementary file4 (DOCX 536 KB)

Supplementary file5 (DOCX 485 KB)

## Data Availability

The data that support the findings of this study are available from the corresponding author upon reasonable request.
