# Supplementary material for: Identification of selection signatures and genetic diversity in the sheep
Source: Trop Anim Health Prod. 2025 Feb 18;57(2):68. doi: 10.1007/s11250-025-04307-9 (PMC11836209; doi:10.1007/s11250-025-04307-9)
Supplement: Supplementary file 1 — Supplementary file1 (DOCX 14 KB) [file 11250_2025_4307_MOESM1_ESM.docx]

**Table S1.** ROH numbers by chromosome

| **Chromosome** | **No. of ROH** |
| --- | --- |
| 1 | 380 |
| 2 | 354 |
| 3 | 318 |
| 6 | 240 |
| 4 | 181 |
| 10 | 174 |
| 5 | 168 |
| 11 | 164 |
| 13 | 158 |
| 9 | 152 |
| 12 | 139 |
| 7 | 139 |
| 18 | 131 |
| 8 | 131 |
| 15 | 128 |
| 16 | 98 |
| 23 | 97 |
| 25 | 96 |
| 26 | 95 |
| 19 | 94 |
| 17 | 93 |
| 14 | 91 |
| 20 | 91 |
| 22 | 91 |
| 21 | 84 |
| 24 | 70 |
