# Supplementary material for: Identification of selection signatures and genetic diversity in the sheep
Source: Trop Anim Health Prod. 2025 Feb 18;57(2):68. doi: 10.1007/s11250-025-04307-9 (PMC11836209; doi:10.1007/s11250-025-04307-9)
Supplement: Supplementary file 2 — Supplementary file2 (DOCX 35 KB) [file 11250_2025_4307_MOESM2_ESM.docx]

**Tabel S2.** QTL information overlapping with genomic regions detected using the iHS and ROH approach

| **iHS Approach** | | | | | | | | | | | | |
| --- | --- | --- | --- | --- | --- | --- | --- | --- | --- | --- | --- | --- |
| **Chr** | **Region Size (bp)** | | **Data Type** | | **Abbreviation** | | **QTL ID** | | **Trait Name** | | **Trait Class** | |
| 1 | 150000000‐150500000 | | QTL | | BONEP | | QTL:14322 | | Carcass bone percentage | | Meat and Carcass | |
|  |  |  | QTL | | FA-C22:5 | | QTL:17216 | | Meat docosapentaenoic acid content | | Meat and Carcass | |
|  |  |  | QTL | | BONE_WT | | QTL:14275 | | Bone weight in carcass | | Meat and Carcass | |
|  |  |  | QTL | | BONE_WT | | QTL:14249 | | Bone weight in carcass | | Meat and Carcass | |
|  |  |  | QTL | | MUSWT | | QTL:14276 | | Muscle weight in carcass | | Meat and Carcass | |
|  |  |  | QTL | | LMYP | | QTL:14278 | | Lean meat yield percentage | | Meat and Carcass | |
|  |  |  | QTL | | FATP | | QTL:14277 | | Carcass fat percentage | | Meat and Carcass | |
|  |  |  | QTL | | FLYD | | QTL:17228 | | Fleece yield | | Wool | |
|  |  |  | QTL | | FA-C20:5 | | QTL:17215 | | Meat eicosapentaenoic acid content | | Meat and Carcass | |
|  |  |  | QTL | | PUFA | | QTL:17221 | | Meat polyunsaturated fatty acid content | | Meat and Carcass | |
| 9 | 31500000-32000000 | | QTL | | HCWT | | QTL:14290 | | Hot carcass weight | | Meat and Carcass | |
|  |  |  | QTL | | UYC | | QTL:14012 | | Useful yield content | | Milk | |
|  |  |  | QTL | | SAOS | | QTL:17194 | | Salmonella abortusovis susceptibility | | Health | |
|  |  |  | QTL | | RUMPW | | QTL:122128 | | Rump width | | Exterior | |
|  |  |  | QTL | | CHWDT | | QTL:122129 | | Chest width | | Exterior | |
|  |  |  | QTL | | LMA | | QTL:14323 | | Longissimus muscle area | | Meat and Carcass | |
|  |  |  | QTL | | LMA | | QTL:14291 | | Longissimus muscle area | | Meat and Carcass | |
|  |  |  | QTL | | MUSWT | | QTL:14289 | | Muscle weight in carcass | | Meat and Carcass | |
| 9 | 33000000-33500000 | | QTL | | HCWT | | QTL:14290 | | Hot carcass weight | | Meat and Carcass | |
|  |  |  | QTL | | SAOS | | QTL:17194 | | Salmonella abortusovis susceptibility | | Health | |
|  |  |  | QTL | | LMA | | QTL:14323 | | Longissimus muscle area | | Meat and Carcass | |
|  |  |  | QTL | | LMA | | QTL:14291 | | Longissimus muscle area | | Meat and Carcass | |
|  |  |  | QTL | | MUSWT | | QTL:14289 | | Muscle weight in carcass | | Meat and Carcass | |
| 9 | 34500000-35000000 | | QTL | | HCWT | | QTL:14290 | | Hot carcass weight | | Meat and Carcass | |
|  |  |  | QTL | | SAOS | | QTL:17194 | | Salmonella abortusovis susceptibility | | Health | |
|  |  |  | QTL | | LMA | | QTL:14323 | | Longissimus muscle area | | Meat and Carcass | |
|  |  |  | Association | | LS | | QTL:211617 | | Total lambs born | | Reproduction | |
|  |  |  | QTL | | LMA | | QTL:14291 | | Longissimus muscle area | | Meat and Carcass | |
|  |  |  | QTL | | MUSWT | | QTL:14289 | | Muscle weight in carcass | | Meat and Carcass | |
| 9 | 37000000-37500000 | | QTL | | HCWT | | QTL:14290 | | Hot carcass weight | | Meat and Carcass | |
|  |  |  | QTL | | LMA | | QTL:14323 | | Longissimus muscle area | | Meat and Carcass | |
|  |  |  | QTL | | LMA | | QTL:14291 | | Longissimus muscle area | | Meat and Carcass | |
|  |  |  | QTL | | MUSWT | | QTL:14289 | | Muscle weight in carcass | | Meat and Carcass | |
| 9 | 39000000-39500000 | | QTL | | HCWT | | QTL:14290 | | Hot carcass weight | | Meat and Carcass | |
|  |  |  | QTL | | LMA | | QTL:14323 | | Longissimus muscle area | | Meat and Carcass | |
|  |  |  | QTL | | LMA | | QTL:14291 | | Longissimus muscle area | | Meat and Carcass | |
|  |  |  | QTL | | MUSWT | | QTL:14289 | | Muscle weight in carcass | | Meat and Carcass | |
| 9 | 43000000-43500000 | | QTL | | HCWT | | QTL:14290 | | Hot carcass weight | | Meat and Carcass | |
|  |  |  | QTL | | LMA | | QTL:14323 | | Longissimus muscle area | | Meat and Carcass | |
|  |  |  | QTL | | LMA | | QTL:14291 | | Longissimus muscle area | | Meat and Carcass | |
|  |  |  | QTL | | MUSWT | | QTL:14289 | | Muscle weight in carcass | | Meat and Carcass | |
| 9 | 48500000-49000000 | | QTL | | HCWT | | QTL:14290 | | Hot carcass weight | | Meat and Carcass | |
|  |  |  | QTL | | LMA | | QTL:14324 | | Longissimus muscle area | | Meat and Carcass | |
|  |  |  | QTL | | LMA | | QTL:14291 | | Longissimus muscle area | | Meat and Carcass | |
|  |  |  | QTL | | MUSWT | | QTL:14263 | | Muscle weight in carcass | | Meat and Carcass | |
|  |  |  | QTL | | MUSWT | | QTL:14289 | | Muscle weight in carcass | | Meat and Carcass | |
| 11 | 29500000-30500000 | | QTL | | LATRICH_2 | | QTL:12901 | | Trichostrongylus adult and larva count | | Health | |
|  |  |  | QTL | | INTFAT | | QTL:14298 | | İnternal fat amount | | Meat and Carcass | |
|  |  |  | QTL | | MYPERS | | QTL:16018 | | Milk yield persistency | | Milk | |
|  |  |  | QTL | | MY | | QTL:16017 | | Milk yield | | Milk | |
|  |  |  | QTL | | PY | | QTL:16019 | | Milk protein yield | | Milk | |
|  |  |  | QTL | | HCWT | | QTL:14296 | | Hot carcass weight | | Meat and Carcass | |
|  |  |  | QTL | | BW | | QTL:14297 | | Body weight | | Production | |
|  |  |  | QTL | | JAWL | | QTL:13802 | | Jaw length | | Meat and Carcass | |
|  |  |  | QTL | | MFA-C12:0 | | QTL:13894 | | Milk lauric acid content | | Milk | |
|  |  |  | QTL | | MFA-C10:0 | | QTL:13898 | | Milk capric acid content | | Milk | |
|  |  |  | QTL | | MFA-C10:0 | | QTL:13899 | | Milk capric acid content | | Milk | |
|  |  |  | QTL | | MFA-C12:0 | | QTL:13901 | | Milk lauric acid content | | Milk | |
|  |  |  | QTL | | JAWL | | QTL:14178 | | Jaw length | | Meat and Carcass | |
|  |  |  | QTL | | MPUFA | | QTL:13896 | | Milk polyunsaturated fatty acid content | | Milk | |
| 12 | 39000000-39500000 | | QTL | | MY | | QTL:14148 | | Milk yield | | Milk | |
|  |  |  | QTL | | FECGEN | | QTL:256942 | | Fecal egg count | | Health | |
|  |  |  | Association | | MCHC | | QTL:257591 | | Mean corpuscular hemoglobin concentration | | Health | |
|  |  |  | Association | | MCH | | QTL:257582 | | Mean corpuscular hemoglobin content | | Health | |
|  |  |  | QTL | | FATP | | QTL:14299 | | Carcass fat percentage | | Meat and Carcass | |
|  |  |  | QTL | | LMYP | | QTL:14300 | | Lean meat yield percentage | | Meat and Carcass | |
| **ROH Approach** | | | | | | | | | | | | |
| **Chr** | | **Region Size (bp)** | | **Data Type** | | **Abbreviation** | | **QTL ID** | | **Trait Name** | | **Trait Class** |
| 6 | | 40311379-42930840 | | QTL | | MUSWT | | QTL:14333 | | Muscle weight in carcass | | Meat and Carcass |
|  |  |  |  | Association | | BOA | | QTL:95749 | | Bone area | | Meat and Carcass |
|  |  |  |  | Association | | TOTBONE | | QTL:95764 | | Total bone | | Production |
|  |  |  |  | Association | | BOA | | QTL:95750 | | Bone area | | Meat and Carcass |
|  |  |  |  | QTL | | MFDIAM | | QTL:14017 | | Mean fiber diameter | | Wool |
|  |  |  |  | QTL | | FECGEN | | QTL:16024 | | Fecal egg count | | Health |
|  |  |  |  | QTL | | FECZ | | QTL:13843 | | Facial eczema susceptibility | | Health |
|  |  |  |  | QTL | | FATP | | QTL:14285 | | Carcass fat percentage | | Meat and Carcass |
|  |  |  |  | Association | | ADG | | QTL:193075 | | Average daily gain | | Production |
|  |  |  |  | Association | | KLEIB | | QTL:193089 | | Kleiber ratio | | Production |
|  |  |  |  | QTL | | HCWT | | QTL:14283 | | Hot carcass weight | | Meat and Carcass |
|  |  |  |  | Association | | LS | | 130449 | | [Total lambs born](https://www.animalgenome.org/cgi-bin/QTLdb/OA/qtrait?trait_ID=3036) | | Reproduction |
|  |  |  |  | QTL | | BW | | QTL:14284 | | Body weight | | Production |
|  |  |  |  | QTL | | HCWT | | QTL:14260 | | Hot carcass weight | | Meat and Carcass |
|  |  |  |  | QTL | | BW | | QTL:14261 | | Body weight | | Production |
|  |  |  |  | QTL | | LMYP | | QTL:14286 | | Lean meat yield percentage | | Meat and Carcass |
|  |  |  |  | QTL | | FATWT | | QTL:14282 | | Fat weight in carcass | | Meat and Carcass |
|  |  |  |  | QTL | | FATWT | | QTL:14332 | | Fat weight in carcass | | Meat and Carcass |
| 11 | | 26956590-27412690 | | QTL | | LATRICH_2 | | QTL:12901 | | Trichostrongylus adult and larva count | | Health |
|  |  |  |  | QTL | | INTFAT | | QTL:14298 | | İnternal fat amount | | Meat and Carcass |
|  |  |  |  | QTL | | MYPERS | | QTL:16018 | | Milk yield persistency | | Milk |
|  |  |  |  | QTL | | MY | | QTL:16017 | | Milk yield | | Milk |
|  |  |  |  | QTL | | PY | | QTL:16019 | | Milk protein yield | | Milk |
|  |  |  |  | Association | | HEIGHT | | QTL:136166 | | Body height | | Production |
|  |  |  |  | QTL | | HCWT | | QTL:14296 | | Hot carcass weight | | Meat and Carcass |
|  |  |  |  | QTL | | BW | | QTL:14297 | | Body weight | | Production |
|  |  |  |  | QTL | | MPUFA | | QTL:13896 | | Milk polyunsaturated fatty acid content | | Milk |
| 11 | | 27654920-30218516 | | Association | | CURDF | | QTL:224770 | | Curd firmness | | Milk |
|  |  |  |  | QTL | | LATRICH_2 | | QTL:12901 | | Trichostrongylus adult and larva count | | Health |
|  |  |  |  | Association | | ADG | | QTL:57792 | | Average daily gain | | Production |
|  |  |  |  | QTL | | INTFAT | | QTL:14298 | | İnternal fat amount | | Meat and Carcass |
|  |  |  |  | QTL | | MYPERS | | QTL:16018 | | Milk yield persistency | | Milk |
|  |  |  |  | QTL | | MY | | QTL:16017 | | Milk yield | | Milk |
|  |  |  |  | QTL | | PY | | QTL:16019 | | Milk protein yield | | Milk |
|  |  |  |  | Association | | MY_180d | | QTL:169331 | | Milk Yield {180d} | | Milk |
|  |  |  |  | Association | | MFY_180d | | QTL:169330 | | Milk fat yield {180d} | | Milk |
|  |  |  |  | QTL | | HCWT | | QTL:14296 | | Hot carcass weight | | Meat and Carcass |
|  |  |  |  | QTL | | BW | | QTL:14297 | | Body weight | | Production |
|  |  |  |  | QTL | | JAWL | | QTL:13802 | | Jaw length | | Meat and Carcass |
|  |  |  |  | QTL | | MFA-C12:0 | | QTL:13894 | | Milk lauric acid content | | Milk |
|  |  |  |  | QTL | | MFA-C10:0 | | QTL:13898 | | Milk capric acid content | | Milk |
|  |  |  |  | QTL | | MFA-C10:0 | | QTL:13899 | | Milk capric acid content | | Milk |
|  |  |  |  | QTL | | MFA-C12:0 | | QTL:13901 | | Milk lauric acid content | | Milk |
|  |  |  |  | QTL | | JAWL | | QTL:14178 | | Jaw length | | Meat and Carcass |
|  |  |  |  | QTL | | MPUFA | | QTL:13896 | | Milk polyunsaturated fatty acid content | | Milk |
