# Supplementary material for: Identification of selection signatures and genetic diversity in the sheep
Source: Trop Anim Health Prod. 2025 Feb 18;57(2):68. doi: 10.1007/s11250-025-04307-9 (PMC11836209; doi:10.1007/s11250-025-04307-9)
Supplement: Supplementary file 3 — Supplementary file3 (DOCX 14 KB) [file 11250_2025_4307_MOESM3_ESM.docx]

**Tablo S3.** QTL information overlapping with genomic regions detected using the iHS approach

| **Trait Class** | **No** | **%** |
| --- | --- | --- |
| Meat and Carcass | 59 | 53.15% |
| Wool | 2 | 1.80% |
| Milk | 25 | 22.52% |
| Production | 10 | 9.01% |
| Exterior | 2 | 1.80% |
| Health | 11 | 9.91% |
| Reproduction | 2 | 1.80% |
| Total | 111 | 100.00% |
