# Supplementary material for: Identification of selection signatures and genetic diversity in the sheep
Source: Trop Anim Health Prod. 2025 Feb 18;57(2):68. doi: 10.1007/s11250-025-04307-9 (PMC11836209; doi:10.1007/s11250-025-04307-9)
Supplement: Supplementary file 4 — Supplementary file4 (DOCX 536 KB) [file 11250_2025_4307_MOESM4_ESM.docx]

**Tabel S4.** Table of top 10 significant p-values for GO Biological Process 2021, GO Molecular Function 2021, GO Cellular Component 2021 and KEGG 2021 Human

| **GO Biological Process 2021** | | |
| --- | --- | --- |
| **Term** | **P-value** | **Overlap genes** |
| heart morphogenesis (GO:0003007) | 7.78e-03 | SOX17, CHD7 |
| regulation of neurotransmitter receptor activity (GO:0099601) | 9.26e-03 | CRH, SHISA6 |
| protein retention in Golgi apparatus (GO:0045053) | 1.24e-02 | VPS13D |
| glutamate catabolic process (GO:0006538) | 1.24e-02 | ADHFE1 |
| regulation of mitophagy (GO:1901524) | 1.24e-02 | VPS13D |
| cardiac cell fate commitment (GO:0060911) | 1.24e-02 | SOX17 |
| cardiac endothelial cell differentiation (GO:0003348) | 1.24e-02 | SOX17 |
| endocardial cell differentiation (GO:0060956) | 1.24e-02 | SOX17 |
| endodermal cell fate specification (GO:0001714) | 1.24e-02 | SOX17 |
| regulation of extracellular exosome assembly (GO:1903551) | 1.49e-02 | SDCBP |
| 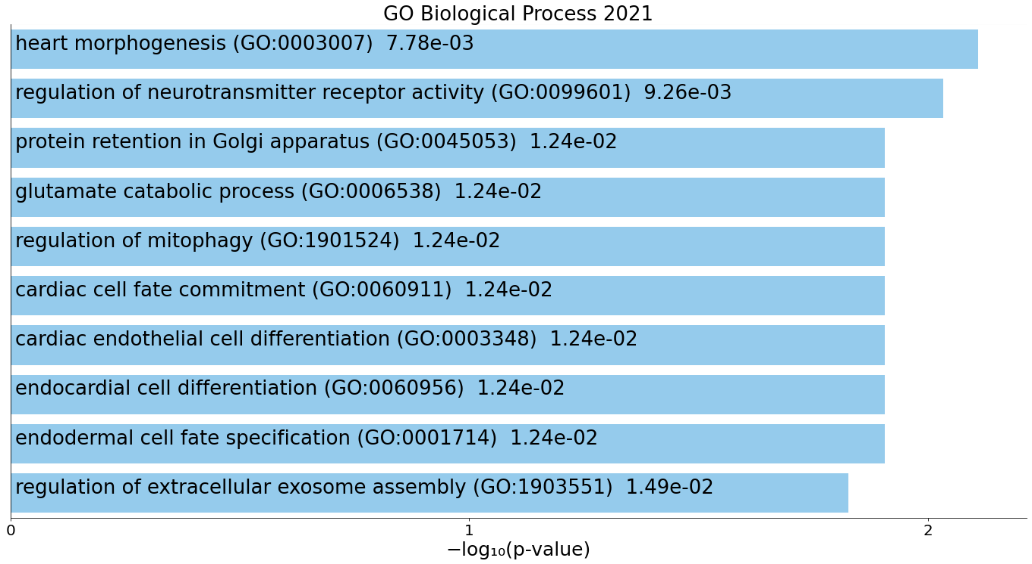 | | |
| **GO Molecular Function 2021** | | |
| **Term** | **P-value** | **Overlap genes** |
| syndecan binding (GO:0045545) | 1.24e-02 | SDCBP |
| alcohol dehydrogenase [NAD(P)+] activity (GO:0018455) | 1.74e-02 | ADHFE1 |
| alcohol dehydrogenase (NAD+) activity (GO:0004022) | 2.23e-02 | ADHFE1 |
| 5S rrna binding (GO:0008097) | 2.23e-02 | RRS1 |
| ionotropic glutamate receptor binding (GO:0035255) | 2.47e-02 | SHISA6 |
| MAP kinase kinase activity (GO:0004708) | 3.69e-02 | MAP2K4 |
| glutamate receptor binding (GO:0035254) | 4.17e-02 | SHISA6 |
| NAD-retinol dehydrogenase activity (GO:0004745) | 4.65e-02 | DHRS3 |
| 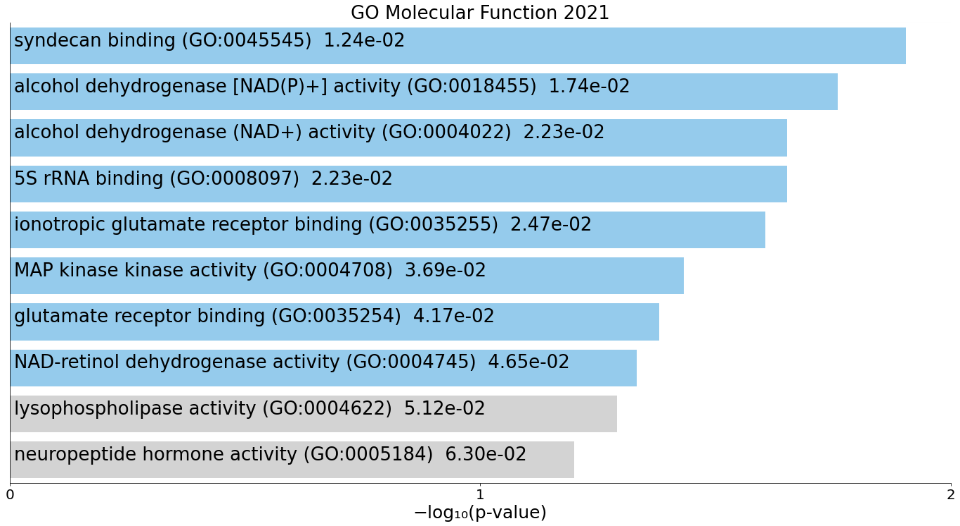 | | |
| **GO Cellular Component 2021** | | |
| **Term** | **P-value** | **Overlap genes** |
| asymmetric, glutamatergic, excitatory synapse (GO:0098985) | 1.49e-02 | SHISA6 |
| nuclear chromosome (GO:0000228) | 1.83e-02 | RRS1, SPIDR |
| dendritic spine membrane (GO:0032591) | 1.98e-02 | SHISA6 |
| proton-transporting V-type atpase, V1 domain (GO:0033180) | 1.98e-02 | ATP6V1H |
| vacuolar proton-transporting V-type atpase, V1 domain (GO:0000221) | 1.98e-02 | ATP6V1H |
| vacuolar proton-transporting V-type atpase complex (GO:0016471) | 4.65e-02 | ATP6V1H |
| AMPA glutamate receptor complex (GO:0032281) | 4.89e-02 | SHISA6 |
| condensed nuclear chromosome (GO:0000794) | 4.89e-02 | RRS1 |
| 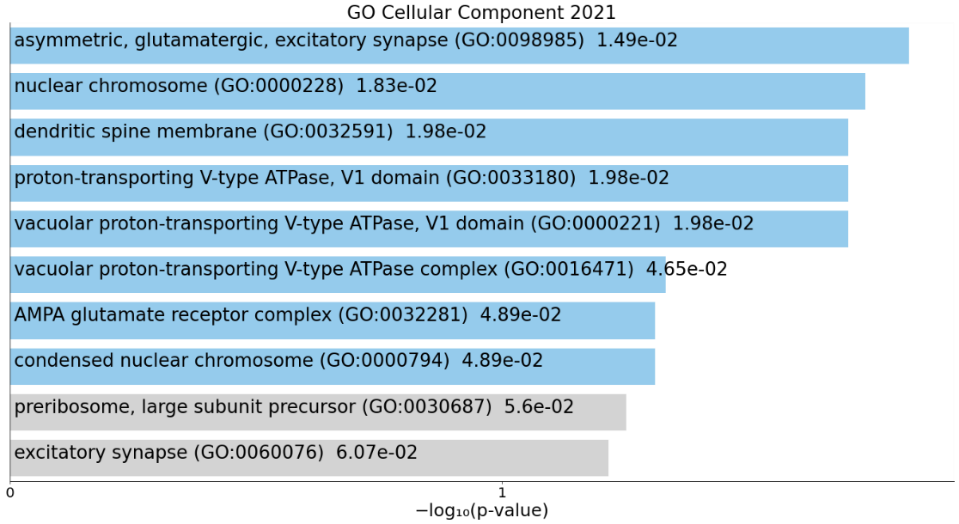 | | |
| **KEGG 2021 Human** | | |
| **Term** | **P-value** | **Overlap genes** |
| Epithelial cell signaling in Helicobacter pylori infection | 1.33e-02 | MAP2K4, ATP6V1H |
| 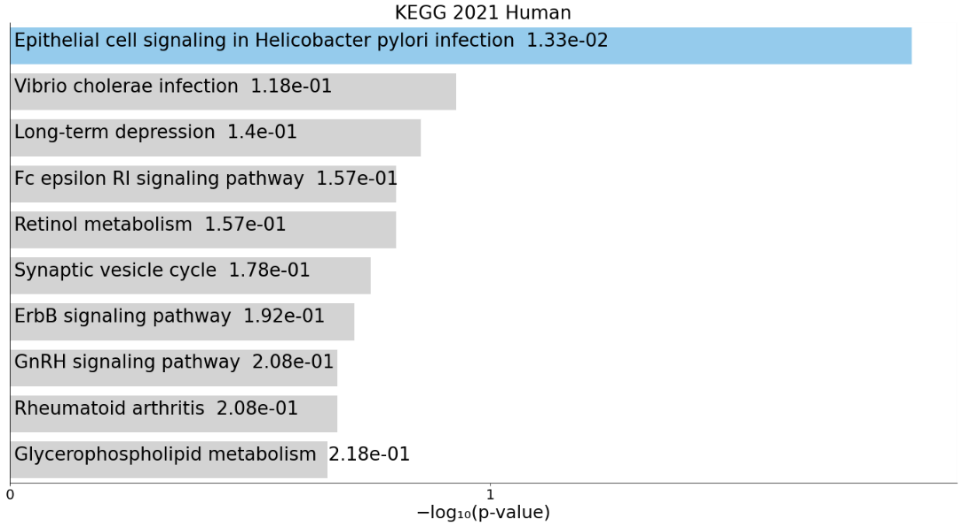 | | |
