# Supplementary material for: Identification of selection signatures and genetic diversity in the sheep
Source: Trop Anim Health Prod. 2025 Feb 18;57(2):68. doi: 10.1007/s11250-025-04307-9 (PMC11836209; doi:10.1007/s11250-025-04307-9)
Supplement: Supplementary file 5 — Supplementary file5 (DOCX 485 KB) [file 11250_2025_4307_MOESM5_ESM.docx]

**Table S5.** Top 10 significant p-values for GO Biological Process 2021, GO Molecular Function 2021, GO Cellular Component 2021 and KEGG 2021 Human

| **GO Biological Process 2021** | | |
| --- | --- | --- |
| **Term** | **P-value** | **Overlap genes** |
| hepoxilin biosynthetic process (GO:0051122) | 7.50e-06 | ALOX12B, ALOXE3, ALOX15B |
| hepoxilin metabolic process (GO:0051121) | 7.50e-06 | ALOX12B, ALOXE3, ALOX15B |
| actin-myosin filament sliding (GO:0033275) | 2.63e-05 | MYH2, MYH3, MYH8, MYH4 |
| muscle filament sliding (GO:0030049) | 2.63e-05 | MYH2, MYH3, MYH8, MYH4 |
| lipoxygenase pathway (GO:0019372) | 3.98e-05 | ALOX12B, ALOX15B, ALOXE3 |
| linoleic acid metabolic process (GO:0043651) | 1.14e-04 | ALOX12B, ALOX15B, ALOXE3 |
| muscle contraction (GO:0006936) | 3.08e-04 | MYH2, MYH3, MYH8, MYH13, MYH4 |
| long-chain fatty acid biosynthetic process (GO:0042759) | 3.38e-04 | ALOX12B, ALOXE3, ALOX15B |
| lipid oxidation (GO:0034440) | 1.55e-03 | ALOX12B, ALOX15B |
| unsaturated fatty acid metabolic process (GO:0033559) | 1.91e-03 | ALOX12B, ALOXE3, ALOX15B |
| 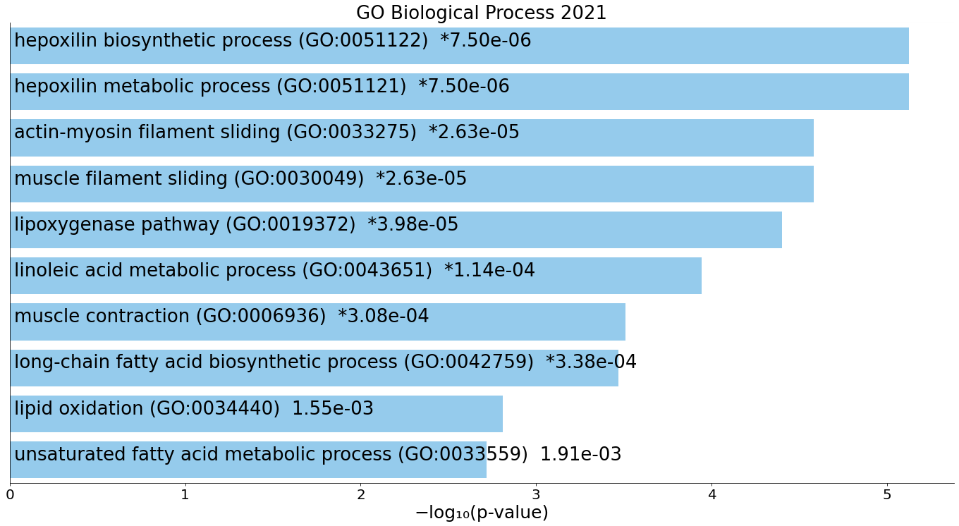 | | |
| **GO Molecular Function 2021** | | |
| **Term** | **P-value** | **Overlap genes** |
| oxidoreductase activity, acting on single donors with incorporation of molecular oxygen, incorporation of two atoms of oxygen (GO:0016702) | 7.08e-05 | ALOX12B, ALOX15B, ALOXE3 |
| 1-phosphatidylinositol-3-kinase regulator activity (GO:0046935) | 2.36e-03 | PIK3R6, PIK3R5 |
| phosphatidylinositol 3-kinase regulator activity (GO:0035014) | 3.33e-03 | PIK3R6, PIK3R5 |
| protein serine/threonine/tyrosine kinase activity (GO:0004712) | 7.17e-03 | MAP2K4, AURKB |
| potassium channel regulator activity (GO:0015459) | 2.18e-02 | KCNIP4, KCNAB3 |
| ADP-ribose diphosphatase activity (GO:0047631) | 2.25e-02 | ADPRM |
| guanylate cyclase activator activity (GO:0030250) | 2.25e-02 | RCVRN |
| histone demethylase activity (H3-K27 specific) (GO:0071558) | 2.25e-02 | KDM6B |
| beta-galactosidase activity (GO:0004565) | 2.25e-02 | GBA3 |
| histone serine kinase activity (GO:0035174) | 2.70e-02 | AURKB |
| 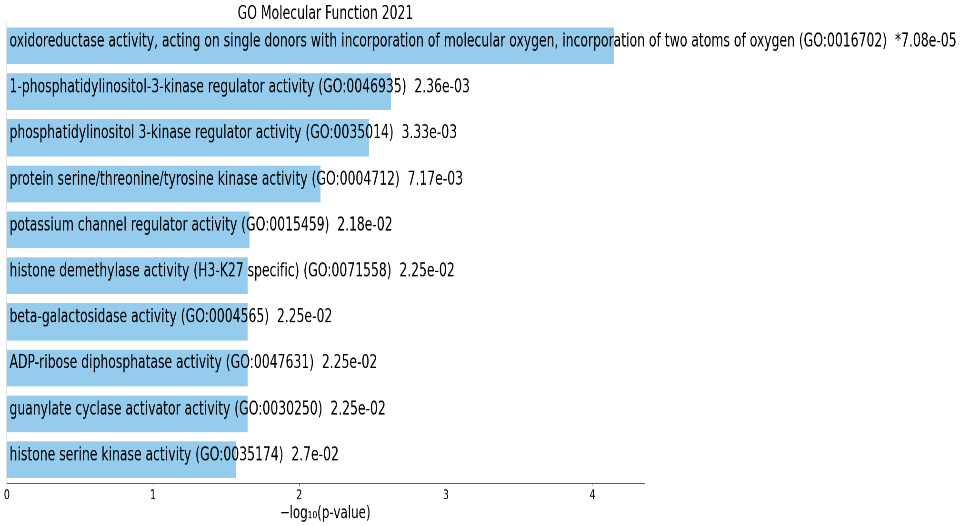 | | |
| **GO Cellular Component 2021** | | |
| **Term** | **P-value** | **Overlap genes** |
| muscle myosin complex (GO:0005859) | 3.40e-11 | MYH1, MYH2, MYH3, MYH13, MYH8, MYH4 |
| phosphatidylinositol 3-kinase complex, class I (GO:0097651) | 1.98e-04 | PIK3R6, PIK3R5 |
| myosin filament (GO:0032982) | 2.97e-04 | MYH8, MYH10 |
| myofibril (GO:0030016) | 6.53e-03 | MYH2, MYH4 |
| clathrin-sculpted monoamine transport vesicle (GO:0070081) | 2.23e-02 | VAMP2 |
| clathrin-sculpted monoamine transport vesicle membrane (GO:0070083) | 2.23e-02 | VAMP2 |
| asymmetric, glutamatergic, excitatory synapse (GO:0098985) | 2.67e-02 | SHISA6 |
| zymogen granule (GO:0042588) | 2.67e-02 | VAMP2 |
| zymogen granule membrane (GO:0042589) | 2.67e-02 | VAMP2 |
| 9+2 motile cilium (GO:0097729) | 2.72e-02 | DNAH2, DNAH9 |
| 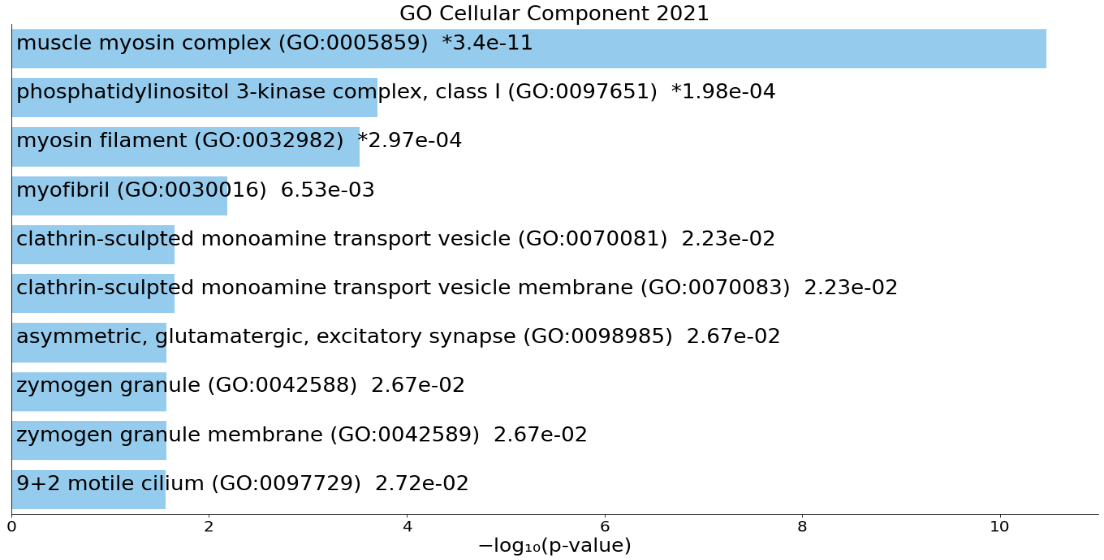 | | |
| **KEGG 2021 Human** | | |
| **Term** | **P-value** | **Overlap genes** |
| Phototransduction | 7.17e-03 | RCVRN, GUCY2D |
| SNARE interactions in vesicular transport | 9.86e-03 | STX8, VAMP2 |
| Purine metabolism | 2.11e-02 | GUCY2D, ADPRM, PFAS |
| Arachidonic acid metabolism | 3.15e-02 | ALOX12B, ALOX15B |
| 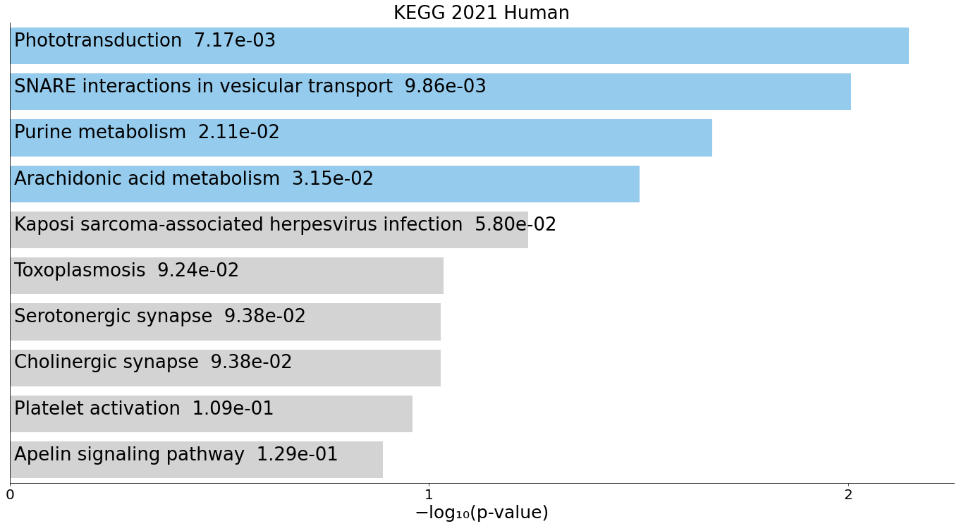 | | |
